# Effectiveness of interventions aimed at improving physical and psychological outcomes of fall-related injuries in people with dementia: a narrative systematic review

**DOI:** 10.1186/s13643-018-0697-6

**Published:** 2018-02-20

**Authors:** Shannon Robalino, Sarange B. Nyakang’o, Fiona R. Beyer, Chris Fox, Louise M. Allan

**Affiliations:** 1 Present Address: Kaiser Permanente Research Affiliates, Evidence-based Practice Center, Center for Health Research, Portland, OR USA; 2 Institute of Health and Society, Newcastle University, Newcastle upon Tyne, UK; 3 Dementia Research Collaborative Norwich Medical School, University of East Anglia, Norwich, UK; 4 Norfolk and Suffolk NHS Foundation Trust, Norwich, UK; 5 Institute of Neuroscience, Newcastle University, Newcastle upon Tyne, UK

**Keywords:** Accidental falls, Dementia, Fractures, Geriatrics, Narrative reviews, Soft tissue injuries

## Abstract

**Background:**

The annual prevalence of falls in people with dementia ranges from 47 to 90%. Falls are a common reason for hospital admission in people with dementia, and there is limited research evidence regarding the care pathways experienced by this population. In addition to immediate management of an injury, prevention of further falls is likely to be an important part of any successful intervention.

This review aims to assess the effectiveness of interventions for improving the physical and psychological wellbeing of people with dementia who have sustained a fall-related injury.

**Methods:**

Systematic review methodologies were employed utilising searches across multiple databases (MEDLINE, CENTRAL, Health Management Information Consortium, EMBASE, CINAHL, Web of Science, Allied and Complementary Medicine Database, and Physiotherapy Evidence Database (PEDro)) and citation chaining. Studies including people with a known diagnosis of dementia living in the community and who present at health services with a fall, with or without injury, were included. Outcomes of interest included mobility, recurrent falls, activities of daily living, length of hospital stay, and post-discharge residence. Results were independently reviewed and quality assessed by two researchers, and data extracted using a customised form. A narrative synthesis was performed due to heterogeneity of the included studies.

**Results:**

Seven studies were included. Interventions clustered into three broad categories: multidisciplinary in-hospital post-surgical geriatric assessment; pharmaceuticals; and multifactorial assessment. Multidisciplinary care and early mobilisation showed short-term improvements for some outcomes. Only an annual administration of zoledronic acid showed long-term reduction in recurrent falls.

**Conclusions:**

Due to high heterogeneity across the studies, definitive conclusions could not be reached. Most post-fall interventions were not aimed at patients with dementia and have shown little efficacy regardless of cognitive status. Minor improvements to some quality of life indicators were shown, but these were generally not statistically significant. Conclusions were also limited due to most studies addressing hip fracture; the interventions provided for this type of injury may not be suitable for other types of fractures or soft tissue injuries, or for use in primary care.

**Systematic review registration:**

PROSPERO CRD42016029565.

**Electronic supplementary material:**

The online version of this article (10.1186/s13643-018-0697-6) contains supplementary material, which is available to authorized users.

## Background

In 2014, an estimated 850,000 people were living with dementia in the United Kingdom [1], 70% of whom were living in their own homes [2]. The annual prevalence of falls in people with dementia (PWD) ranges from 47 to 90% [3, 4], depending on dementia subtype; those living in their own home sustain almost 10 times more incident falls than those with a cognitive impairment, and their falls are more likely to be injurious [4]. Dementia is an independent risk factor for experiencing a serious injury (e.g. fracture or head injury) related to a fall [4]. A recent Australian study indicates the most common fracture-related injury in dementia patients is to the hip followed by the trunk [5]. Other common serious injuries not resulting in a fracture in PWD include those to the head and neck, lower limbs, and traumatic brain injury [5]. Compared to other patients, PWD are typically at greater risk of hospitalisation, prolonged hospital stays, increased care demands, and incomplete recovery after a fall [6].

Dementia is generally initially assessed with cognitive screening tests (e.g. the Mini Mental State Exam (MMSE)) and performance of Activities of Daily Living (ADLs) (e.g. bathing, dressing, mobility). Depending on the outcomes of these assessments, a diagnosis may be given ranging from mild to moderate to severe. Mild forms of dementia indicate the person can perform most ADLs without assistance while a person with severe dementia may be completely dependent on a carer to perform all ADLs.

There is limited research evidence regarding the care pathways experienced by PWD presenting with a fall-related injury, although it is known that falls are a common reason for hospital admission in people with dementia [7] with a third of those sustaining only soft tissue injuries, not fractures [5, 8]. Comorbid factors are more likely to be present in people with dementia [9] and include an underlying acute medical cause of the fall, delirium, inability to mobilise, and carer stress or lack of ability to support them after the fall. Up to 40% of people presenting to the emergency services (ES) have a cognitive disorder [10], which is a barrier to good emergency care thereby resulting in preventable admissions. A recent review found that the evidence underpinning the management of PWD in the ES reflects expert opinion rather than controlled trials [10], and there is a paucity of evidence-based pathways for hospital care [11]. Staff often perceives PWD as less capable of rehabilitation due to lack of person-centred supportive strategies [12]. PWD presenting to ES and those admitted to hospital are therefore currently managed using services not designed to meet their needs; we believe a successful intervention would improve care in the ES and may reduce or shorten hospital admissions.

Furthermore, some PWD with fall-related injuries may not present to ES or may present directly to primary care, and a majority of injuries sustained are minor soft tissue injuries which may not require medical attention [4]. A successful intervention must therefore also work for PWD whose care is managed in the community. Improvements in the management of fall-related injuries might reduce further complications for PWD and carers by improving physical recovery, ameliorating fear of falling, and psychological morbidity [13], all of which may lead the person to restrict their mobility. Restriction of mobility results in deconditioning and a cycle of further loss of mobility and frailty.

In addition to immediate management of an injury, prevention of further falls is likely to be an important part of any successful intervention. For older people without dementia, it has been reported that a multifactorial intervention by a specialist falls service will prevent further falls [14, 15]. Although the components of such an intervention are usually directed at known risk factors for falls, these methods have not been shown to be consistently effective in PWD [16] and indeed there are trials which have shown no benefit [17, 18]. The primary reasons for this may be that risk factors for falls may differ in PWD or be more frequent in or specific to dementia, e.g. wandering [19], Parkinsonism [20, 21], severity of cognitive impairment [20], and functional impairment [22].

In summary, PWD have complex needs which are not fully met by existing services. Several recent reviews address fall risk and fall prevention in cognitively impaired older people and PWD [16, 23–26]. The aim of this paper is to perform a systematic review and meta-analysis of the current research evidence for effectiveness of interventions intended to improve the physical and psychological wellbeing of PWD who have sustained a fall-related injury of any kind. The primary outcomes of interest are measures of performance-oriented assessment of mobility (e.g. Tinetti score) and measures of performance in ADLs (e.g. Barthel score). Secondary outcomes of interest are length of hospital stay, place of discharge post-intervention, recurrent fall or injury, and readmission to hospital. We are interested in measures up to 3 months post-intervention though timings are variable based on what is reported in the studies. This review also forms part of a larger study funded by the National Institutes of Health Research on the development of a complex intervention to improve the outcome of fall-related injuries in PWD living in their own homes.

## Methods

### Protocol and registration

The protocol for this review was registered with PROSPERO (CRD42016029565) [27]. The review is described according to PRISMA guidelines [28] and the PRISMA checklist is available in Additional file 1.

### Search strategy and selection criteria

Table 1 provides an overview of the search methods and selection criteria. Databases were searched from inception to the reported search date. An update search in January 2018 of Medline only was conducted due to limited resources and evidence that Medline generally provides the largest yield of included studies [29]. A sample Medline search strategy is available in Additional file 2.

**Table 1:** Search methods including databases and dates of searches, search term facets, limitations, and inclusion/exclusion criteria

Databases searched (inception through November 2015, Medline update January 2018)	MEDLINE, CENTRAL, Health Management Information Consortium, EMBASE, CINAHL, Web of Science, Allied and Complementary Medicine Database, Physiotherapy Evidence Database (PEDro), Clinicaltrials.gov, ISRCTN registry
Search facets and limits*	Facets: dementia, falls and fall-related injuries, and interventions Limits: English language and RCT filters [47] applied when possible
Inclusion criteria	Design: RCT or quasi-experimental Population: PWD living in the community having sustained a fall, with or without injury Intervention: any type of intervention directed at people with dementia who have fallen including multifactorial assessment or intervention Comparisons: usual care Outcomes: measures of performance-oriented assessment of mobility, ADLs, length of hospital stay, place of discharge, recurrent fall or injury, and hospital readmission
Exclusion criteria	Studies not published in English Cognitively intact patient populations or sub-analysis for PWD not done PWD living in care homes only

*See Additional file 2 for sample Medline search strategy

Citation chaining was used for included papers and relevant systematic reviews to identify additional papers of interest. Grey literature was not included due to limited resources. Citations were stored in EndNote.

Following Cochrane principles, two reviewers (SR and FRB) independently screened titles and abstracts to identify potentially relevant papers. Disagreements were resolved through discussion and where necessary with a third reviewer (LMA or CF). For studies taken forward to full-text review, two reviewers (SR and FRB) screened the full text to determine inclusion or exclusion of the study; discrepancies were resolved through discussion or by a third reviewer (LMA or CF).

### Data extraction

Data were extracted using a bespoke Excel form by one reviewer (SR) and were checked by a second (SBN). Discrepancies were resolved through discussion. Data collected included details of the study population (e.g. MMSE score), setting (e.g. ward), the intervention (e.g. care team, services used) and comparator, and outcomes (e.g. mobility, length of hospital stay) measured at baseline and follow-up. For studies that satisfied inclusion criteria but whose reported outcomes were incomplete, authors were contacted three times via email to provide additional data.

### Quality assessment

The Cochrane Risk of Bias tool [30] was used to assess methodological quality within and across studies. Two reviewers (SR and SBN) independently assessed each study. Studies were assessed across five domains of bias and deemed to be at high-, low-, or unclear risk of bias on each domain. Disagreements were discussed and where necessary a third reviewer (FRB) was consulted.

### Data synthesis

We planned to perform a meta-analysis, but study heterogeneity made this impractical. Few studies measured the same outcomes, and even where these outcomes were similar enough to warrant comparison, their measurement tools varied, thereby precluding a valid statistical analysis. Therefore, we carried out a narrative synthesis broadly categorising studies by intervention and presenting detailed results by outcomes of interest.

## Results

### Study selection

**Fig. 1:**
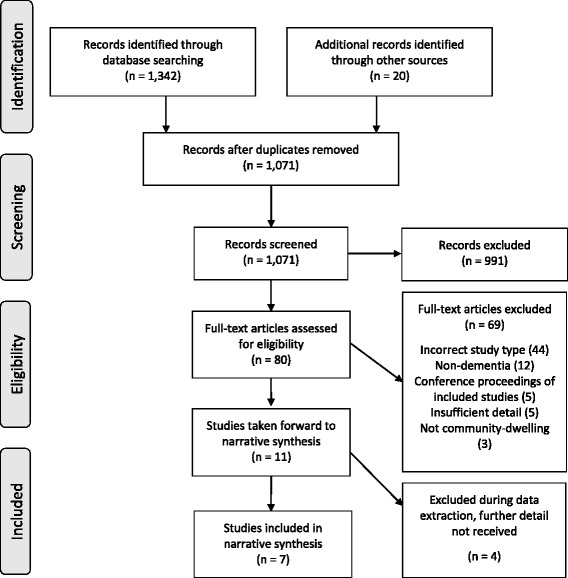
PRISMA flow diagram (Moher et al. 2009) [28]

The PRISMA flow diagram in Fig. 1 provides a summary of the study selection process. Seven studies were selected for inclusion.

### Study characteristics

This review included six RCTs [18, 31–35] and one quasi-experimental study [36] (see Table 2 for detailed characteristics). Studies were conducted in the United Kingdom [18, 35], Canada [36], Finland [31], Norway [34], Sweden [33], and one which included 24 countries [32]. All studies included both cognitively intact and diagnosed dementia patients (*n* = 1061) with the exception of Shaw [18], which recruited patients on the basis of a Mini Mental State Exam (MMSE) score of < 24 indicating at least mild dementia was present.

**Table 2:** Summary of included studies

Study	Study design	Setting (location)	Participants (PWD)	Interventions	Comparator	Outcomes		Effectiveness (authors’ comments)
						Functional outcome measure	Other outcomes	
Huusko et al. (2000) [31]	RCT	Hospital (Finland)	*N* = 141 I/C: 78/63 Mean age (years): 80 I/C: 80/80	Intensive rehabilitation within a geriatric ward—physiotherapy twice/day, early ambulation, self-motivation, daily living aids, occupational therapy, individualised patient and family counselling, home visits	Rehabilitation in a local hospital	n/a	Post-discharge residence; length of hospital stay; mortality	The intervention shortened the length of hospital stay in patients with mild or moderate dementia.
Kennie et al. (1988) [35]	RCT	Hospital (United Kingdom)	*N* = 51 I/C: 20/31 Median age (years): not reported I/C: 79/84	Physiotherapy, occupational therapy, orthotic services etc. in a peripheral hospital as well as thrice weekly supervision by a geriatrician	Physiotherapy, occupational therapy, orthotic services, etc. in the orthopaedic admission ward	Katz ADL index^a^	Length of hospital stay; post-discharge residence	Geriatric rehabilitative care shortened the length of hospital stays and increased the chances of regaining functional independence and independent life among elderly women with hip fractures.
McGilton et al. (2013) [36]	Quasi-experimental	Hospital (Canada)	*N* = 47 I/C: 23/24^b^ Mean age (years): not reported I/C: 82.5/80.1^c^	Patient centred rehabilitation model targeting patients with cognitive impairment (PCRM-CI)—rehabilitation management, dementia management, delirium prevention, education and support for healthcare providers on providing patient-centred care, education, and support for caregivers	Usual care—rehabilitation management	Functional Independence Measure Motor Subscale (FIMM)	Length of stay; post-discharge residence^d^	No difference in mobility gains, but the intervention increased the likelihood of returning home after discharge.
Prieto-Alhambra et al. (2013) [32]	RCT	Unclear (multiple countries^e^)	*N* = 350 I/C: 168/182 Mean age (years): 78.9	Annual 5 mg IV zoledronic acid	Placebo IV infusion		*Time to first clinical fracture; time to death	Findings support the use of zoledronic acid (bisphosphonates) in cognitively impaired patients with osteoporotic fractures.
Shaw et al. (2003) [18]	RCT	Accident and Emergency (A&E) departments (United Kingdom)	*N* = 246 I/C: 118/128 Mean age (years): Not reported I/C: 84/84	Multifactorial assessment and intervention—medical, cardiovascular, physiotherapy and occupational therapy assessment and management of untreated problems, pharmacotherapy, supervised home-based exercise, and home hazard modification	Conventional care	Gait score; balance score	Number of falls in the first year after intervention; number of falls; time to first fall; injury rates; fall-related attendance at A&E; fall-related hospital admissions; environmental risk factors; cardiovascular risk factors; mortality	The intervention was not shown to be effective in preventing falls in persons with dementia and CI presenting to the A&E after a fall.
Stenvall et al. (2012) [33]	RCT	Hospital (Sweden)	*N* = 64 I/C: 28/36 Mean age: not reported I/C: 81/83.2	Comprehensive geriatric assessments and rehabilitation—prevention, detection, and treatment of postoperative complications, and early mobilisation	Conventional postoperative routines	Swedish Clinical Outcomes Variables (S-COVS); Use of walking aids; ADL staircase	Living situation; re-admissions; in-hospital days after discharge	The intervention resulted in fewer postop complications among patients with dementia; it also improved recovery in their p-ADL^f^ performance and walking ability.
Watne et al. (2014) [34]	RCT	Hospital (Norway)	*N* = 162 I/C: 80/82 Mean age (years): not reported I/C: 84/85	Comprehensive geriatric assessment (CGA) in the acute geriatric ward—multidisciplinary meetings, medication reviews, early and intensive mobilisation, optimised nutrition, and early discharge planning	Treatment in the orthopaedic ward and early mobilisation	Barthel ADL Index; Nottingham Extended ADL Index (NEADL); mobility; SPPB	Preoperative delirium; severity of delirium; length of stay; mortality; place of residence; weight changes	The intervention was not found to improve cognitive function 4 months after surgery, but it had a positive impact on mobility.

^a^Reported only for the median patient, therefore unusable

^b^Based on non-published data

^c^This was for whole study population, not exclusively those with CI

^d^A&E–Accident and Emergency Department

^e^Canada, USA, Argentina, Brazil, Colombia, Guatemala, Peru, Austria, Belgium, Czech Republic, Denmark, Finland, France, Greece, Norway, Poland, Russia, Slovakia, Spain, Switzerland, Sweden, Turkey, United Kingdom, Hong Kong

^f^Personal/primary ADL

Participants’ cognitive status was assessed using the MMSE [18, 31, 33, 34, 36] or the Short Portable Mental Status Questionnaire (SPMSQ) [32, 35]. Only one study included a clinical diagnosis of dementia [33]. Most patients in the included studies presented with, and were treated for, hip fracture. Six of the seven studies had patients aged ≥ 65 years (range 78 to 84 years); most of the patients in the studies were female.

Most of the interventions took place in a hospital [31, 33–36], and one in an A&E department [18]. For one study this was unclear, but appeared to be a clinical setting due to the nature of the intervention [32].

The interventions can be considered in three broad categories:

1. Multidisciplinary in-hospital post-surgical geriatric assessment (five studies [31, 33–36]): these varied in terms of the type of ward (e.g. geriatric versus orthopaedic), mix of staff from multiple disciplines, and components of the intervention. All studies in this group included a core team of a geriatrician, nurse, occupational therapist, and physiotherapist. The studies also included a variety of other staff that were either part of the core team or available depending on the needs of the patient. For example, one study included a social worker as a core team member [31] while Watne et al. [34] had a social worker available as needed. Other staff included a dietician [33], neuropsychologist [31], and a general practitioner (GP) [31, 35]. The actual components of the assessments and interventions also differed, but all assessments and interventions occurred in the hospital setting. For example, early discharge planning was conducted in three studies [31, 34, 36], post-discharge home visits in two [31, 33], and weekly team meetings in three [31, 33, 35]. Full details of the assessments and interventions across these studies can be found in Additional file 3.
2. Pharmaceutical: one study [32] administered an annual dose of intravenous zoledronic acid to participants in an attempt to reduce recurrent falls and further fractures by improving bone health.
3. Multifactorial assessment and intervention: one study [18] performed multifactorial assessments and interventions in patients presenting at an A&E Department post-fall. The multifactorial assessment involved a multidisciplinary team similar to the in-hospital geriatric assessment and followed up with risk assessments in patients’ homes. Patients were offered a variety of interventions based on the risk assessments such as home-based exercise, home hazard modification, medication review, and optical correction by an optician.

None of the studies reported psychological outcomes.

### Synthesis of results

Reported outcomes were highly variable with little overlap in terms of the outcomes measured or the tools used to measure them. Most studies reported place of residence after discharge and a measure of either mobility or ADLs. Table 3 provides a summary view of the studies grouped by our categorisation and outcomes of interest reported.

**Table 3:** Summary table of interventions and reported outcomes of interest

Studies (grouped by broad intervention)	Mobility	Recurrent fall, injury, or other fall-related	Activities of daily living	Length of hospital stay	Place of residence following discharge	Readmission to hospital
Multidisciplinary in-hospital post-surgical geriatric assessment						
Huusko 2000 [31]				✓	✓	
Stenvall 2012 [33]	✓	✓	✓		✓	
Watne 2014 [34]	✓		✓	✓	✓	✓
Kennie 1988 [35]			✓	✓	✓	
McGilton 2013 [36]	✓				✓	
Pharmaceuticals						
Prieto-Alhambra 2014 [32]		✓				
Multifactorial assessment and intervention						
Shaw 2003 [18]	✓	✓				✓

#### Mobility

Four studies reported different measures of mobility following the intervention [18, 33, 34, 36], of which three reported limited improvement or retention of mobility in the intervention group compared to control. Those studies utilising multidisciplinary in-hospital post-surgical geriatric assessment [33, 34, 36] found short-term improvements in gait, but long-term improvements were either not reported or proved statistically insignificant. It should be noted that studies used different mobility scales, and these scales have relatively little overlap in components measured.

#### Recurrent fall, injury, or other fall-related outcome

Three studies reported recurrent falls post-intervention [18, 32, 33], of which only one [33] reported a reduction in in-patient falls in the treatment group (4%) compared to control (31%, *p* = 0.006), although there was no difference in new fractures. A second study [18] reported no difference in the number of patients with falls, the median number of falls, or the median number of weeks before first recurrent fall. The final study [32] found no difference in falls for PWD, but reported a reduction in recurrent fractures at 6 months in the cognitively impaired patients.

#### Activities of daily living

Three studies reported on post-intervention ADLs [33–35] utilising four different tools: the Nottingham Extended ADL Index (NEADL); the Barthel Activities of Daily Living (BADL); personal/primary ADL (P-ADL); and the staircase-ADL. These four scales have limited overlap with only two items common among them (feeding and transferring).

These studies all used multidisciplinary in-hospital post-surgical geriatric assessment and intervention and reported ADLs post-intervention; however, the results are mixed and therefore inconclusive. Two of the studies [34, 35] did not provide baseline ADL assessments though both stated some improvements to those in the cognitively impaired groups. The third study [33] found a larger proportion of those in the treatment group had regained pre-fall ADL levels at 12 months post-intervention (*p* = 0.027).

#### Length of hospital stay

Three studies measured length of hospital stay [31, 34, 35]. All three studies used multidisciplinary in-hospital post-surgical geriatric assessment as an intervention, but the components of those services were variable. They provided some indication that multidisciplinary in-hospital post-surgical geriatric assessment and intervention decreases length of stay for those with mild or moderate dementia.

One study [31] reported length of hospital stay categorised by MMSE scores.^1^ Those in the mild dementia group had a median length of stay of 29 days (range 16–138) and 46 days (range 10–365) for the intervention and control groups, respectively (*p* = 0.002). For moderate dementia, the median length of stay was 47 days (range 10–365) and 147 days (range 18–365) for the intervention and control groups, respectively (*p* = 0.042). The authors therefore concluded that their intervention could reduce length of stay for patients with mild or moderate dementia. However, for severe dementia, the difference in length of stay between the groups was not significant (*p* = 0.902).

Similarly, Kennie et al. [35] reported length of stay based on SPMSQ score at study entry in clusters of general mental status and reported a shorter length of stay in all treatment groups, regardless of mental state.

The intervention group in Watne et al. [34] had a significantly longer median length of stay, 11 days (IQR 6–14) versus 8 days (IQR 3–10) in the control group (*p* ≤ 0.001).

#### Place of residence following discharge

Five studies reported on place of discharge following the intervention [31, 33–36], but the results were contradictory. All five studies utilised multidisciplinary in-hospital post-surgical geriatric assessment. Three studies [31, 35, 36] found patients with moderate dementia in the treatment group were more likely to return to independent living. The remaining studies [33, 34] showed no significant difference in independent living between treatment and control groups regardless of severity of dementia.

#### Readmission to hospital

Readmissions to hospital related to the index fall were reported by two studies [18, 34]. Neither study showed a statistically significant difference in readmission rates between the treatment and control groups with dementia at 4- and 12-months.

### Risk of bias

Table 4 includes both individual study scores and an overall risk of bias score across studies. Studies generally had a low risk of selection bias, and high risk of performance and detection biases due to difficulties in blinding participants and/or personnel to interventions and outcomes – a common scenario with complex interventions. The risk of bias for attrition and reporting were less clear. Most studies included additional biases such as small numbers of included patients with dementia [33] and low or under-recruitment into the study [18, 34] which may have made management of random error difficult. No studies were excluded based on these assessments of risk of bias. Risks of bias forms are available in Additional file 4.

**Table 4:** Risk of bias within and across studies

	Random sequence generation (selection bias)	Allocation concealment (selection bias)	Blinding of participants and personnel (performance bias)	Blinding of outcome assessment (detection bias)	Incomplete outcome data (attrition bias)	Selective reporting (reporting bias)	Other bias
Huusko et al. (2000) [31]	+	+	–	–	+	?	?
Kennie et al. (1988) [35]	+	+	–	–	?	?	?
McGilton et al. (2013) [36]	–	–	–	–	?	?	–
Prieto-Alhambra et al. (2014) [32]	+	+	+	+	–	+	–
Shaw et al. (2003) [18]	+	?	–	–	+	?	?
Stenvall et al. (2012) [33]	?	+	–	+	+	?	–
Watne et al. (2014) [34]	+	+	–	+	+	+	?
Overall score	+	+	–	–	?	?	?

Key: + low risk of bias; − high risk of bias;? unclear risk of bias

## Discussion

### Summary of evidence

This body of evidence assessing the effectiveness of interventions to improve outcomes for PWD who fall comprised different interventions, reported multiple different outcomes, and included people with cognitive impairment as well as those diagnosed with dementia, making it impossible to pool and difficult to summarise. The quality of evidence was mixed and the results across the studies conflicted even when similar interventions were utilised.

Notably, there were variations in the use of the term ‘comprehensive geriatric assessment’ (CGA) [33, 34], especially with regards to the types of core staff delivering the intervention. Additionally, key differences occurred in several areas, some thought to have some effect on outcomes, such as the frequency of multidisciplinary team meetings (daily versus twice per week), discharge planning, post-discharge in-home follow-up [14, 22], falls assessment and prevention, and medication management [37, 38]. This may be contextual, a result of national, regional or local practices in ageing care, or it may be an indication of a gradually evolving definition of the process itself. Current evidence suggests that CGA is likely to benefit older people hospitalised with acute conditions due to these services generally providing a multidimensional, multidisciplinary approach which includes the identification of medical, social and functional needs, as well as the development of an integrated and co-ordinated care plan to address those needs [39, 40]. While CGA has an accepted clinical definition [41], it is known that this interpretation varies widely [42]. The question of whether there is a need for adaptation of CGA for people with dementia has not been addressed.

Nonetheless, several of the studies which focused on providing comprehensive, multidisciplinary in-hospital post-surgical care showed better outcomes for the treatment group. Outcomes were generally short-term, less than 6-months, if they provided any improvement on usual care. In the few instances where it was possible to more directly compare outcomes (e.g. ADLs, length of stay) the results were inconclusive. For example, those in the treatment group in Watne et al. [34] had a significantly longer median length of stay despite statistically insignificant pre-surgical waiting times. We suspect this is likely due to the variance of health care personnel involved, and the actual assessments and interventions used.

Generally, the earlier the patient is mobilised, the better the outcome with regards to reduced length of stay and discharge to independent living. Patients with mild- and moderate dementia also showed better outcomes than those with more severe dementia.

The only intervention which showed a long-term (> 1 year) reduction in the number of incident falls was the annual administration of intravenous zoledronic acid [32].

### Strengths and limitations

This review followed established review methodologies including comprehensive searching for evidence and independent risk of bias assessment.

Four studies that otherwise met the inclusion criteria for this review could not be included [43–46]. The authors of these studies were unsuccessfully contacted either to clarify reported results or to provide sub-group data where it was reported to be available. Three of the studies [43, 45, 46] also used multidisciplinary in-hospital post-surgical geriatric assessment which showed improvements in some outcomes within their treatment groups, regardless of mental status.

Due to the heterogeneity of the interventions and outcomes measured, we were unable to perform a meta-analysis. However, it appears that the use of multidisciplinary assessment and intervention shows some improvement in some post-discharge outcomes.

There were three large gaps in the evidence base. Firstly, most of the population presented with hip fracture so interventions may not be applicable to other types of injury, e.g. soft tissue or other types of fracture. Given that most injuries are not fractures and may still be associated with adverse outcomes, there is a need for further research which includes patients whose injury is not a hip fracture. Secondly, and unsurprisingly, given the predominance of interventions for hip fracture, there was no evidence to guide how fall-related injuries should be managed in primary care. Finally, the studies did not show evidence of any particular adaptation of the approach, enhancement of the skills, or composition of multidisciplinary teams given that they were working with a different population from that of older people without a cognitive impairment. Additionally, most of the interventions were not aimed at patients with known dementia; sub-group analysis was used to report the effects of general interventions on this group.

### Future directions

The ability to work with PWD is considered to be a core skill for older people’s services, but it may be that further enhancement of the multidisciplinary team both in terms of adapting approach and enhancing skills would lead to further benefits. Further research should target those with a clinical diagnosis of dementia and other types of injury besides hip fracture, and study how the approach of a multidisciplinary team should be adapted to meet the needs of people with dementia.

## Conclusions

Few studies have investigated the effectiveness of interventions specifically for PWD, and those we found assessed different interventions and reported various outcomes, making it difficult to draw firm conclusions. Most post-fall interventions aimed at patients with dementia have shown little efficacy. Minor improvements to some quality of life indicators were shown, but these were generally not statistically significant. Multidisciplinary in-hospital post-surgical assessment and intervention showed the most benefit because patients were discharged earlier and returned to living independently. Intravenous zoledronic acid appears to benefit cognitively impaired individuals who are expected to live beyond 6 months post-fracture by reducing recurrence of fracture. There is a need to design RCTs to address all types of injury in people with dementia. Such trials need to consider the views of PWD and their carers with respect to the design and adaptability of the intervention to the person with dementia and also in determining important outcomes to be measured.

## Additional files

Additional file 1: PRISMA Checklist. (PDF 331 kb)

Additional file 2: Medline (OVID) sample search strategy. (PDF 83 kb)

Additional file 3: Full summary table of interventions utilised in geratric assessment. (PDF 191 kb)

Additional file 4: Risk of bias assessment within individual studies. (PDF 71 kb)

## Abbreviations

A&E: Accident and emergency
ADL: Activities of daily living
BADL: Barthel Activities of Daily Living
CGA: Comprehensive geriatric assessment
I/C: Intervention/control
IQR: Interquartile range
MMSE: Mini Mental State Exam
NEADL: Nottingham Extended Activities of Daily Living Index
P-ADL: Personal/Primary Activities of Daily Living
PWD: People with dementia
SPMSQ: Short Portable Mental Status Questionnaire

## Footnotes

1 MMSE scores are generally broken down in the following ranges: 0–9 = severe dementia, 10–19 = moderate dementia, 20–23 = mild dementia, 24+ = fully cognitively intact. However, Huusko et al. [29] reported the scores as 0–11, 12–17, 18–23, and 24–30.
