# Supplementary material for: Effectiveness of interventions aimed at improving physical and psychological outcomes of fall-related injuries in people with dementia: a narrative systematic review
Source: Syst Rev. 2018 Feb 20;7:31. doi: 10.1186/s13643-018-0697-6 (PMC5819703; doi:10.1186/s13643-018-0697-6)
Supplement: Supplementary file 2 — Medline (OVID) sample search strategy. (PDF 83 kb) [file 13643_2018_697_MOESM2_ESM.pdf]

Additional File 2

Example search strategy from OVID MEDLINE

1. exp dementia/
2. exp Supranuclear Palsy, Progressive/ or exp Hydrocephalus, Normal Pressure/
3. (Dementia? or Amentia? or Alzheimer*).ti,ab,hw,kw.
4. ((Creutzfeldt-Jakob or huntington? or kluver-bucy or lewy-bod* or (lewy adj2 bod*)) adj3 (Syndrome or disease or disorder or dementia?)).ti,ab,hw,kw.
5. ((normal adj2 hydrocephalus) or (supranuclear adj1 palsy) or (picks adj1 (disorder or disease))).ti,ab,hw,kw.
6. or/1-5
7. ((Accidental* adj3 Fall?) or Falls or Fall-related or Fracture? or ((bone? or hip or femur or tibia or arm?) adj3 broken)).mp.
8. (fall* adj3 injur*).mp.
9. exp fractures, bone/
10. accidental falls/
11. or/7-10
12. exp accident prevention/
13. (preventi* or prevent).mp.
14. intervention?.mp.
15. exp Rehabilitation/
16. rehabilitat*.mp.
17. exp Nutrition Therapy/
18. ((nutrition* or ergonomic or exercise or occupational or physical) adj3 (support* or therap*)).mp.
19. physiotherap*.mp.
20. (improv* adj5 (outcome? or care)).mp.
21. management.mp.
22. ((psycho* or physical* or mobility) adj5 (outcome? or improv*)).mp.
23. (decreas* adj2 risk?).mp.
24. ((improv* or increas*) adj5 (social* or participation or independence or activit* or well?being or QOL or (quality adj2 life))).mp.
25. exp Activities of Daily Living/
26. ((multifactorial or multicomponent or multidisciplinary) adj3 (team? or assessment or intervention?)).mp.
27. recovery.mp.
28. HRQoL.mp.
29. or/12-28
30. 6 and 11 and 29
