# Supplementary material for: Effectiveness of interventions aimed at improving physical and psychological outcomes of fall-related injuries in people with dementia: a narrative systematic review
Source: Syst Rev. 2018 Feb 20;7:31. doi: 10.1186/s13643-018-0697-6 (PMC5819703; doi:10.1186/s13643-018-0697-6)
Supplement: Supplementary file 3 — Full summary table of interventions utilised in geratric assessment. (PDF 191 kb) [file 13643_2018_697_MOESM3_ESM.pdf]

Additional File 3

Full summary table of interventions utilised in geriatric assessment

	Study						
	Huusko et al. (2000) [1]	McGilton et al. (2013) [2]	Prieto-Alhambra et al. (2013) [3]	Shaw et al. (2003) [4]	Stenvall et al. (2012) [5]	Watne et al. (2014) [6]	Kennie et al. (1988) [7]
**Staff**[1]							
**A&E consultants**				✓[2]			
**Dietician**					✓		
**General practitioner (GP)**	✓			✓			✓
**Geriatrician**	✓				✓	✓	✓
**Neurologist**	✓*						
**Neuropsychologist**	✓						
**Nurse**	✓			✓	✓	✓	
**Nutritionist**						✓*	
**Occupational therapist**	✓			✓	✓	✓	✓
**Optician/ophthalmologist**							
**Orthopaedic specialist**							✓*
**Pastor**							
**Physical medicine consultant**	✓*						
**Physiotherapist**	✓			✓	✓	✓	✓
**Psychiatrist**	✓*						
**Social worker**	✓					✓*	
**Speech therapist**							
**Trauma team**							

[1] Comprehensive geriatric assessment requires a team comprising: a doctor/specialist physician, specialist nurse, senior social worker, physiotherapist and occupational therapist

*These staff may not have been part of the main team but were on call to assist when needed

[2] Assisted with the screening of patients

<u>**Intervention**</u>							
**Site of intervention**	Geriatric ward	Inpatient rehabilitation unit	Multicentre	A&E department; patients' homes	Geriatric ward	Acute geriatric ward	Orthopaedic beds
**Comprehensive Geriatric Assessment (CGA)**					✓	✓	
**Bedside assessments**		✓					
**Case-finding**							
**Daily team meetings**						✓	
**Early discharge planning**	✓	✓				✓	
**Follow-up assessments**			✓		✓	✓	
**HCP[3] Training**		✓					
**In-hospital follow-up**							
**Multidisciplinary evaluation**	✓			✓	✓	✓	✓
**Post-discharge home visits**	✓			✓	✓		
**Pre-discharge home visits**	✓[4]						
**Weekly team meetings**	✓				✓ (X2)		✓
<u>**Domain 1: Physical medical conditions**</u>							
**Bladder re-training[4]**		✓					
**Blood transfusion**							
**Blood tests**							
**Clinical advice and recommendations**	✓						
**Correction of physiological disturbances**						✓	
**Daily oral supplements**			✓[5]			✓	
**Decubitus risk**					✓		

[3] Health Care Professional
[4] If the team thought it necessary
[5] Calcium, vitamin D

**Drug treatment**	✓			✓		
**Elimination**						
**Fatigue assessment**						
**Fracture prevention**					✓	
**Hydration**						
**Medical assessment**					✓	
**Nutrition**					✓	✓
**Optical correction**				✓		
**Osteoporosis assessment**						✓
**Pre-operative electrocardiogram**						
**Pain assessment**						✓
**Pain management[4]**		✓			✓	✓
**Radiographs**			✓			
**Review of drug regimen**				✓		✓
**Sleep interventions[4]**		✓				
**Somatic health assessment**						
**Speech therapy**	✓					
**UTI screening/ treatment**					✓	
**<u>Domain 2: Mental health conditions</u>**						
**Anxiety assessment**						
**Cognitive assessment**						
**Delirium prevention**		✓			✓	✓
**Dementia management**		✓[6]				
**Depression assessment**				✓		
**Mental health assessment**						
**Patient counselling**	✓					
**Psychogeriatric assessment**				✓		
**<u>Domain 3: Functioning</u>**						
**Daily increase of ambulation**		✓				

[6] If required, using the REAP model: Relate well, Environment modification, Abilities-focused care, Personhood

**distance using the lowest level of assistance/aids**							
**Daily living aids**	✓						
**Early mobilization and rehabilitation**	✓				✓	✓	
**Evaluation of ADL**					✓		
**Falls assessment**					✓		
**Falls prevention**					✓		
**Family counselling**	✓						
**Functional assessment**					✓		
**Help with use of appliances**	✓						
**Hip range of motion**		✓					
**Home hazard modification**				✓			
**Home-based exercise program**				✓			
**Increasing independence in basic activities of daily living**		✓					
**Individualised rehabilitation planning**							
**Lower extremity strengthening**		✓					
**Occupational therapy**	✓[7]	✓		✓			✓
**Orthotics**				✓			✓
**Physiotherapy**	✓	✓		✓			✓
**Training**	✓						
**Walking aids**	✓			✓			
**<u>Domain 4: Social circumstances</u>**							
**Caregiver training**		✓					
**Social support services**							

[7] Only for patients who would benefit from it

Supplemental material references

1 Huusko TM, Karppi P, Avikainen V, Kautiainen H, Sulkava R. Randomised, clinically controlled trial of intensive geriatric rehabilitation in patients with hip fracture: subgroup analysis of patients with dementia. BMJ. 2000;321:1107-11.

2 McGilton KS, Davis AM, Naglie G, Mahomed N, Flannery J, Jaglal S, et al. Evaluation of patient-centered rehabilitation model targeting older persons with a hip fracture, including those with cognitive impairment. BMC Geriatr. 2013;13:136.

3 Prieto-Alhambra D, Judge A, Arden NK, Cooper C, Lyles KW, Javaid MK. Fracture prevention in patients with cognitive impairment presenting with a hip fracture: Secondary analysis of data from the HORIZON Recurrent Fracture Trial. Osteoporos Int. 2014;25:77-83.

4 Shaw FE, Bond J, Richardson DA, Dawson P, Steen IN, McKeith IG, et al. Multifactorial intervention after a fall in older people with cognitive impairment and dementia presenting to the accident and emergency department: randomised controlled trial. BMJ. 2003;326:73.

5 Stenvall M, Berggren M, Lundstrom M, Gustafson Y, Olofsson B. A multidisciplinary intervention program improved the outcome after hip fracture for people with dementia-Subgroup analyses of a randomized controlled trial. Arch Gerontol Geriatr. 2012;54:e284-e9.

6 Watne LO, Torbergsen AC, Conroy S, Engedal K, Frihagen F, Hjorthaug GA, et al. The effect of a pre- and postoperative orthogeriatric service on cognitive function in patients with hip fracture: randomized controlled trial (Oslo Orthogeriatric Trial). BMC Med. 2014;12:63.

7 Kennie DC, Reid J, Richardson IR, Kiamari AA, Kelt C. Effectiveness of geriatric rehabilitative care after fractures of the proximal femur in elderly women - a randomized clinical-trial. BMJ. 1988;297:1083-6.
