# Supplementary material for: Effectiveness of interventions aimed at improving physical and psychological outcomes of fall-related injuries in people with dementia: a narrative systematic review
Source: Syst Rev. 2018 Feb 20;7:31. doi: 10.1186/s13643-018-0697-6 (PMC5819703; doi:10.1186/s13643-018-0697-6)
Supplement: Supplementary file 4 — Risk of bias assessment within individual studies. (PDF 71 kb) [file 13643_2018_697_MOESM4_ESM.pdf]

Additional File 4

Risk of Bias assessment within individual studies

Author/Year	Random sequence generation (selection bias) - H/L/U	Support for judgement	Allocation concealment (selection bias) - H/L/U	Support for judgement	Blinding of participants & personnel (performance bias) - H/L/U	Support for judgement	Blinding of outcome assessment (detection bias) - H/L/U	Support for judgement	Incomplete outcome data (attrition bias) - H/L/U	Support for judgement	Selective reporting (reporting bias) - H/L/U	Support for judgement	Other bias - H/L/U	Support for judgement
Huusko/2000	Low	allocation sequence was computer generated	Low	and sealed in numbered, opaque envelopes in Helsinki, Finland, by the information technology department of Novartis before the study was started. The envelopes were stored on the orthopaedic ward by the head nurse until patients were randomised	High	We could not blind the staff doing interventions or assessments.<br><br>Patient blinding not mentioned	High	We could not blind the staff doing interventions or assessments.	Low	Eleven patients were later excluded because of a violation of the randomisation criteria, three patients withdrew their consent after randomisation, and three patients were excluded because of a protocol violation. A total of 243 patients were followed. O	Unclear	Can't locate a trial register record.	n/a	n/a
Kennie/1988	Unclear	Can't access reference<br><br>Patients were allocated in random sequence determined before the start of the study after the method of Tukey.	Low	allocation was in sealed envelopes held by a departmental secretary	High	No attempt was made to blind either staff or patients to the fact that the trial was being conducted	High	No attempt was made to blind either staff or patients to the fact that the trial was being conducted	Unclear	Data reported for 108 in trial	Unclear	Can't locate a trial register record.	n/a	n/a
McGilton/2013	High	Not an RCT<br><br>A quasi-experimental design was used to evaluate the PCRM-CI.	High	Not an RCT<br><br>A quasi-experimental design was used to evaluate the PCRM-CI.	High	there was no blinding of patients, collateral informants, or research assistants.	High	there was no blinding of patients, collateral informants, or research assistants.	Low	See additional table provided by McGilton for sub-analysis	Low	Outcomes reported match outcomes in trial register<br><br>https://clinicaltrials.gov/show/NCT01566136	High	the limited sample size provided insufficient power to examine multiple outcomes and interactions among predictors. Third, this study used a quasi-experimental design as it was impossible to randomly assign the patients to the intervention or control grou

Prieto-Alhambra/2014	Low	From: Cathleen S. Colón-Emeric, John Caminis, Theodore T. Suh, Carl F. Pieper, Cheri Janning, Jay Magaziner, Jonathan Adachi, Theresa Rosario-Jansen, Peter Mesenbrink, Zeb D. Horowitz & Kenneth W. Lyles (2004) The HORIZON Recurrent Fracture Trial: design	Low	From: Cathleen S. Colón-Emeric, John Caminis, Theodore T. Suh, Carl F. Pieper, Cheri Janning, Jay Magaziner, Jonathan Adachi, Theresa Rosario-Jansen, Peter Mesenbrink, Zeb D. Horowitz & Kenneth W. Lyles (2004) The HORIZON Recurrent Fracture Trial: design	Low	From: Lyles KW, Colon-Emeric CS, Magaziner JS, Adachi JD, Pieper CF, Mautalen C, Hyldstrup L, Recknor C, Nordsletten L, Moore KA, Lavecchia C, Zhang J, Mesenbrink P, Hodgson PK, Abrams K, Orloff JJ, Horowitz Z, Eriksen EF, Boonen S (2007) Zoledronic acid	Low	From: Lyles KW, Colon-Emeric CS, Magaziner JS, Adachi JD, Pieper CF, Mautalen C, Hyldstrup L, Recknor C, Nordsletten L, Moore KA, Lavecchia C, Zhang J, Mesenbrink P, Hodgson PK, Abrams K, Orloff JJ, Horowitz Z, Eriksen EF, Boonen S (2007) Zoledronic acid	High	Some patients couldn't be included in sub-analysis due to missing data<br><br>"Among a total of 2,127 participants within the HORIZON Recurrent Fracture Trial, data on cognitive status were available for 1,966 (92.4%) patients. Patients with missing SPMSQ data	Low	Outcomes reported match outcomes in published trial protocol<br><br>Cathleen S. Colón-Emeric, John Caminis, Theodore T. Suh, Carl F. Pieper, Cheri Janning, Jay Magaziner, Jonathan Adachi, Theresa Rosario-Jansen, Peter Mesenbrink, Zeb D. Horowitz & Kenneth W. Ly	High	Some patients couldn't be included in sub-analysis due to missing data<br><br>"Among a total of 2,127 participants within the HORIZON Recurrent Fracture Trial, data on cognitive status were available for 1,966 (92.4%) patients. Patients with missing SPMSQ data
Shaw/2003	Low	randomised patients by block randomisation using computer generated random numbers either to assessment plus targeted multifactorial intervention (intervention group) or to assessment plus conventional care (control group).	Low	Group allocation was performed by a researcher who was independent of the recruitment process and blind to baseline interview data	High	No mention of participant blinding<br><br>Data on secondary outcomes, compliance with intervention, treatment received by control group, and objective effects of intervention, were by necessity recorded and coded by members of the study team, who were not blind	High	Data from the postcards (primary outcome) were processed and coded off site by a researcher who was blind to group allocation and otherwise unconnected with the study. Data on secondary outcomes, compliance with intervention, treatment received by control	Low	See table 2<br><br>We report on 274 of the 308 patients; data on initial multifactorial assessment or outcome of falls (diary returns) were not obtained on 34 patients who died (n□24) or withdrew (n□10) shortly after recruitment.<br><br>Overall, 88% of diaries we	Unclear	No details in trials register http://www.isrctn.com/ISRCTN66023158	Unclear	there was relative under-recruitment of participants from the community, and recruitment was from a spe- cific population in a single centre

Stenvall/2012	Unclear	Doesn't appear to be stated	Low	From: Stenvall, M., Olofsson, B., Lundstro¨m, M., Englund, U., Borssen, B., Svensson, O., Nyberg, L., Gustafson, Y., 2007. A multidisciplinary, multifactorial intervention program reduces postoperative falls and injuries after femoral neck fracture. Osteo	High	From: Stenvall, M., Olofsson, B., Lundstro¨m, M., Englund, U., Borssen, B., Svensson, O., Nyberg, L., Gustafson, Y., 2007. A multidisciplinary, multifactorial intervention program reduces postoperative falls and injuries after femoral neck fracture. Osteo	High	Another limitation is that the assessors were not blinded to the allocation group but to minimize the risk of bias a nurse from the orthopedic department carried out the assessments in the intervention group and a nurse from the geriatric department carri	Low	See figure 1	Unclear	Can't locate a trial register record.	High	…the group studied is small since it is a subgroup analysis. This causes power problems so the results should be interpreted with caution.
Watne/2014	Low	Randomization was based on computer-generated random numbers (blocks of variable and unknown size) and was carried out by a statistician (ES) not involved in the clinical service. Randomization was stratified according to whether or not the patients were	Low	Allocation was by sealed, opaque, numbered envelopes.	High	As with all service evaluations, blinding of assessments during hospital stay was impossible and may have introduced bias.<br><br>Follow up visits were carried out four and twelve months after surgery (with a time window of ± three weeks) by study nurses blind	Low	A statistical analysis plan (SAP) was developed (and published online) prior to un-blinding of the data [33]. The primary analysis was carried out blind to allocation by the study statistician (ES).<br><br>Follow up visits were carried out four and twelve month	Low	We also carried out sensitivity analyses including the three moribund patients who were erroneously recruited, and a strict intention to treat analysis with all patients analyzed according to allocation. Missing values for the primary outcome were imputed	Low	Outcomes reported match outcomes in trial register<br><br>https://clinicaltrials.gov/ct2/show/NCT01009268	Unclear	Lack of power calcultoin prior to recruitment - No pre-trial data were available to carry out precise power estimates. Based upon previous experience with the CDR, we judged 300 patients to be sufficient to detect clinically meaningful differences [30]. A
